# Motion behavior of droplets on curved leaf surfaces driven by airflow

**DOI:** 10.3389/fpls.2024.1450831

**Published:** 2024-10-15

**Authors:** Zhou-Ming Gao, Wei Hu, Xiao-Ya Dong, Xiao-Yuan Zhao, Song Wang, Jian Chen, Bai-Jing Qiu

**Affiliations:** Key Laboratory of Modern Agricultural Equipment and Technology, Ministry of Education, Jiangsu University, Zhenjiang, Jiangsu, China

**Keywords:** airflow, droplet, curved leaf surface, critical wind speed, motion behavior

## Abstract

In air-assisted spraying, pesticide droplet retention on crop leaves is key to evaluating spray effectiveness. However, airflow can deform leaves, reducing droplet retention and affecting spray performance. This study used wind tunnels and high-speed cameras to capture leaf deformation at different airflow speeds and the motion of droplets on curved leaf surfaces. The results showed that leaf curvature during bending deformation is generally less than 0.05 mm^-1^. Critical wind speed for droplet movement is negatively correlated with droplet size and leaf curvature, with a 24.8% difference between different leaf curvatures and a 17.5% difference between droplet sizes. The droplet’s dimensionless shape variable is positively correlated with both droplet size and leaf curvature. The maximum shape variable on curved leaves reaches 0.24, with acceleration differences of about 30%, while droplets of different sizes show a maximum shape variable of 0.18 and an acceleration difference of up to 68%. These findings enhance understanding of droplet-leaf interactions and provide insights for improving pesticide efficiency.

## 1 Introduction

The movement of droplets on the surface of leaves is a complex process influenced by the chemical composition of the droplets and the specific characteristics of the leaf surface (Gaskin et al., 2005). The ultimate goal is to maximize the retention of spray droplets and evenly distribute them on crop leaves to enhance the efficacy of pesticides. Currently, research on the interaction between spray droplets and leaves primarily focuses on the behavior of droplets upon impact and their performance after successfully being retained on the leaves. However, it remains unclear whether droplets can successfully remain on leaves under the influence of airflow. This study aims to examine the ability of droplets to resist external wind forces during air-assisted spraying and to reveal the movement behavior of droplets on leaves.

Existing studies have revealed various kinetic behaviors of droplets upon impact with a leaf surface, including adhesion, fragmentation, splashing, and bouncing (Zabkiewicz, 2007). Dorr et al. (2015) investigated the effects of spray Equation and leaf wetting characteristics on droplet behavior. Their results showed that droplet fragmentation increased with size and impact velocity, when droplets hit hydrophilic leaf surfaces, they are more likely to stick or fragment. In contrast, droplets tend to bounce off when they impact hydrophobic surfaces. Wang et al. (2021) investigated the deposition preferences of pesticide droplets on leaves using a boom mounted with three flat fan-shaped hydraulic nozzles positioned 1 m above the leaves, moving at a speed of 1 m/s. The droplets impacted the leaves at a velocity of approximately 2.8 m/s. The study found that the deposition of pesticide droplets on the leaf was primarily influenced by the leaf’s elasticity. Tredenick et al. (2021) further investigated the evaporation behavior of surfactant droplets on plant leaves and synthetic surfaces at tilt angles of 0°, 45°, and 90° using an automatic tilt platform and high-speed camera. The results revealed that the microstructure and chemical properties of plant leaves significantly differ from synthetic surfaces in terms of evaporation behavior. While the tilt angle of the surface does influence evaporation, the concentration of surfactants has a more pronounced effect on droplet evaporation behavior. All of these studies used the method of cutting leaves and affixing them to solid planes, which is a justified approach. However, their treatments of the leaves primarily considered flat or inclined surfaces, overlooking curved surfaces. Natural plant leaves are highly sensitive to minor disturbances such as wind and raindrops (Gart et al., 2015; de Langre, 2019; Ma et al., 2023), which can cause bending and deformation. Therefore, the behavior of droplets on curved leaf surfaces cannot be ignored.

Some researchers have focused on the effect of the curved structure of the leaf on droplet motion. Wang et al. (2005) investigated the motion of droplets on curved surfaces such as leaves, and further achieved an accurate simulation of droplet motion by using a virtual surface and a dynamic contact angle model to generate a stable contact angle. Mayo et al. (2015) investigated the movement of droplets on leaf surfaces with curvature gradients by placing droplets of various sizes near the peak of the leaf surface and observing their movement paths. They discovered that the motion of 0.3 mm diameter spray droplets was primarily driven by the curvature gradient of the leaf surface. However, as the droplet diameter increased to 5 mm, gravity became the dominant force influencing droplet movement. Zheng et al. (2023) investigated the sedimentation behavior of droplets impacting curved rice leaves. They characterized the natural bending of these leaves during growth using the curvature radius. Their research revealed that the curved surface structure of the leaves significantly affects droplet deposition behavior. These studies above examined the curvature gradient and natural bending of the leaf surface, which are influenced by the structural properties and growth mode of the leaf. However, when subjected to external forces like wind, the leaf experiences significant deformations, and the behavior of droplets on such extensively deformed leaves remains unclear.

Current research on the dynamic behavior of fluid ridges on solid substrates under airflow provides valuable insights into the retention of pesticide droplets on plant leaves. Most of these studies rely on theoretical modeling and numerical simulations. For example, Sullivan et al. (2008, 2012) examined the effect of shear stress on the morphology and stability of fluid ridges on inclined and vertical plane substrates. By solving a mathematical model based on the Navier-Stokes equation, they analyzed the effects of shear stress, initial fluid ridge morphology, and surface tension. Their results indicated that strong shear stress can cause fluid ridges to rupture, with stability also influenced by surface tension and initial morphology. Wilson et al. (2011) investigated the dynamic behavior of fluid films and ridges under shear stress on vertical plane substrates, focusing on energy conversion during liquid film rupture. They found that shear stress significantly affects the rupture location, timing, and energy threshold. Paterson et al. (2015) explored the coupling between fluid ridge shape and airflow velocity distribution, revealing that higher airflow velocities have a notable impact on fluid ridge morphology, while ridge stability depends on airflow velocity, surface tension, and initial morphology. Building on their work with planar substrates, Paterson et al. (2014) further investigated the effect of uniform shear stress on the flow of fluid ridges over a horizontal cylindrical surface, finding that the flow pattern depends on both the shear stress and the cylinder’s curvature. Mitchell et al. (2022) explored the unsteady behavior of coating flow on a rotating cylinder under non-cyclonic and cyclic flow conditions, discovering that the interaction between airflow circulation and cylinder rotation speed can lead to complex flow patterns, which in turn affect the final distribution of the coating. These studies collectively demonstrate that airflow exerts a significant influence on the morphology and stability of fluid ridges, making it a crucial factor to consider.

In addition, existing research has shown that aerodynamics significantly impacts the dynamic behavior of droplets on solid substrates (Ding and Spelt 2008; Ding et al., 2010) investigated the critical conditions for three-dimensional droplets to initiate movement on solid surfaces under shear flow, as well as their sliding, detachment, and fracture behaviors. They found that the critical condition for droplet sliding is closely related to the balance between applied shear stress, surface tension, and resistance from contact angle hysteresis. Droplet size and contact angle hysteresis were also found to influence the critical conditions for sliding and eventual detachment. Wilson et al. (2023) studied droplet morphology, movement paths, and detachment processes under varying airflow velocities and fiber spacings using wind tunnel experiments and high-speed imaging. Their results showed that droplet motion and deformation are affected by airflow velocity and interactions between adjacent droplets. Smaller fiber spacings enhance droplet interactions, leading to more complex motion patterns. While the above research primarily focuses on artificial surfaces, plant leaf surfaces naturally possess more complex structures. Therefore, studying the dynamic behavior of droplets on natural plant leaf surfaces can deepen our understanding of the interactions between droplets and leaves.

Oqielat et al. (2011) used the Frangipani leaf surface as the basis for droplet motion, modeling the leaf surface as a triangular element composed of multiple inclined planes, guiding droplet motion through the steepest path. It was found that the small structures and angle changes on the surface of the leaves have a significant impact on the movement path of water droplets. Building on this foundation, Veremieiev et al. (2014) further investigated the flow of biopesticide droplets on inclined plane leaves, focusing on the diffusion, coalescence, and evaporation processes through experiments and numerical simulations. Their research demonstrated that factors such as the leaf microstructure and chemical properties, droplet size, environmental temperature and humidity, and wind speed significantly influence the flow behavior of droplets. The above studies primarily examined the dynamics of droplets on inclined leaves influenced by gravity, yet practical engineering applications must also consider airflow forces. Particularly in airflow-assisted spraying, deposited droplets on the leaf may migrate due to airflow, merging with others along their path to form larger droplets. These larger droplets can be lost in runoff along leaf edges, leading to pesticide wastage, reduced efficacy, and environmental pollution.

In this study, a wind tunnel and high-speed camera system were employed to observe the motion of droplets on curved capsicum leaves under airflow. The critical wind speeds necessary for initiating droplet movement on these curved leaves were determined. A mechanical analysis model considering droplet size and leaf curvature was developed, and the influence of these factors on droplet behavior was analyzed. The ultimate goal is to offer new insights for enhancing pesticide utilization and optimizing decisions related to airflow-assisted spraying.

## 2 Materials and methods

### 2.1 Plants cultivation and sample preparation

**Figure 1:**
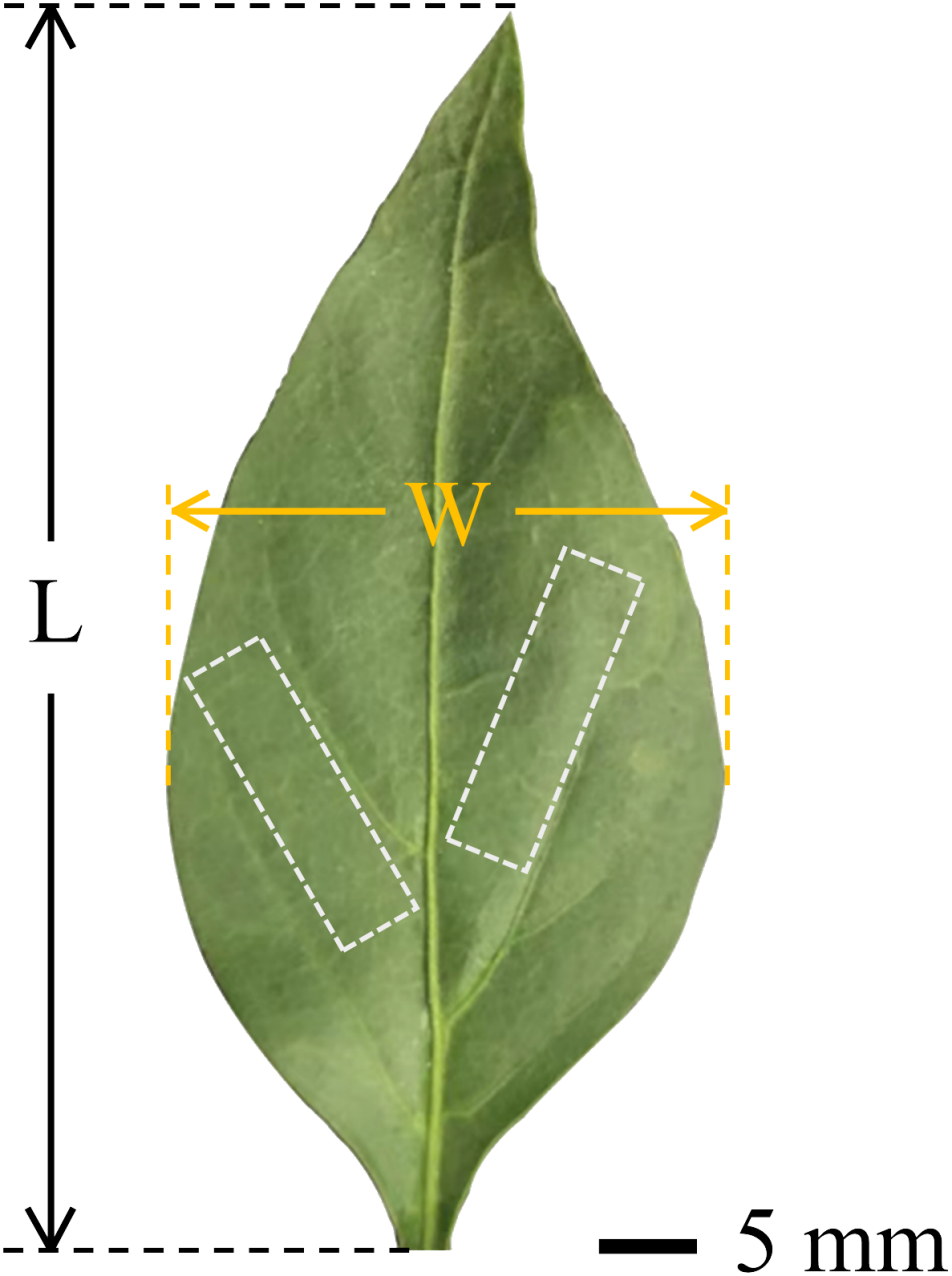
Capsicum leaf morphological and geometric parameters. (The white area is the clipping area).

Capsicum plants (Capsicum annuum L., Tianshuai101) were selected as test samples. Leaves from capsicum plants with a growth cycle of about 3 months were used, with the average leaf area measured by ImageJ software being approximately 1461 mm². The marginal profile of the capsicum leaves was considered elliptical, with their geometry described by leaf length (L) and width (W), as illustrated in **Figure 1**. The leaf size and physical parameters are detailed in **Table 1**.

**Table 1:** Sample size and physical parameters.

Parameter	Unit	Value
Leaf length (L)	mm	58 ± 7
Leaf width (W)	mm	26 ± 5
Leaf area	mm^2^	1461 ± 305
Leaf midrib modulus of elasticity	MPa	8 ± 2
Leaf mesophyll modulus of elasticity	MPa	3 ± 1

Healthy, undamaged, and fully saturated leaves were selected as experimental samples. A sharp blade was used to cut the petiole 10 - 15 mm from the base of the leaf, and the remaining petiole was clamped with a sleeve clamp. The intact leaves were placed vertically at the center of the wind tunnel testing section, with the front of the leaf facing the wind. The movement of the petiole was fully restricted, allowing only the bending deformation of the leaf under airflow to be considered. After successfully capturing the deformation data, the adjacent secondary veins of the capsicum leaves were cut to obtain rectangular leaf sections measuring 6 × 18 mm (marked in white in **Figure 1**). Double-sided tape was used to attach the cut leaf samples smoothly onto a custom-made curved substrate. ensuring the leaves were tightly secured without wrinkles to maintain the accuracy and consistency of subsequent experiments. The entire process was completed within one minute to minimize changes in leaf moisture content due to evaporation caused by the cuts.

### 2.2 Experiment setup

#### 2.2.1 The overall configuration of experiment instruments

**Figure 2:**
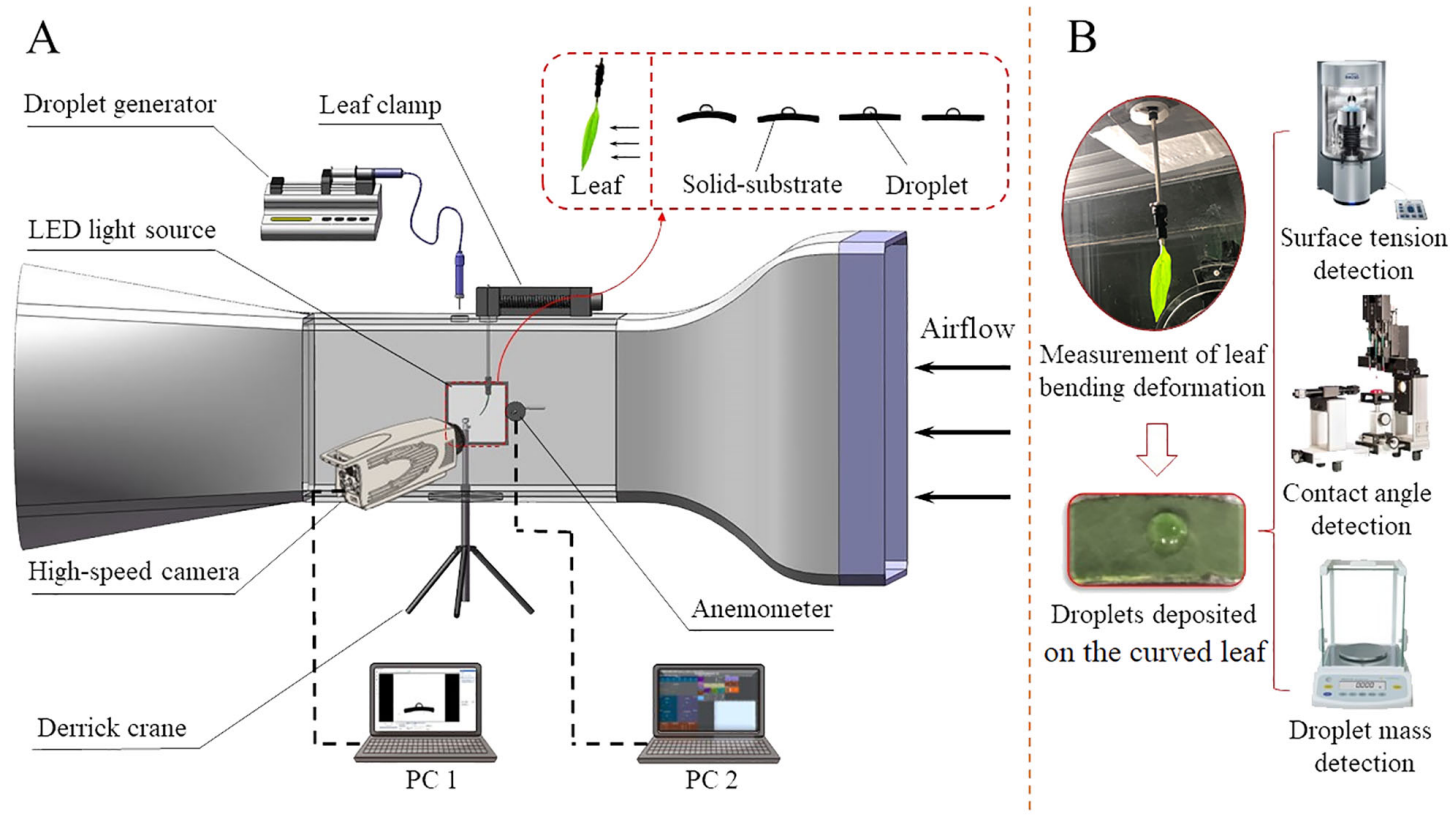
Experimental schematic diagram (arrow indicating airflow direction): **(A)** Experimental platform for leaf and droplet motion behavior; **(B)** Droplet physical property detection platform.

The overall experimental setup is illustrated in **Figure 2**. It includes a droplet physical characteristic detection platform and a test platform for observing the motion behavior of leaves and droplets under airflow. The droplet physical property detection platform comprises a contact angle measuring instrument (OCA 25, Dataphysics, Germany), a surface tension instrument (Kruss K100, Klux, Germany), and a precision analytical balance (BT125D, Kunshan Norbond Electronic Technology Co., Ltd., Suzhou, China). The test platform for leaf and droplet motion behavior under airflow includes a small open wind tunnel, a pitot tube anemometer, a high-speed camera (SH6-109, Shenzhen Shenshi Intelligent Technology Co., Ltd., China), PC (Legion Y7000P, Lenovo Co., Ltd., Beijing, China), a micro injection pump (MDG-100, TSI, USA), a tripod, an LED light source, and homemade leaf clamp and curved substrates with different curvatures. (For detailed information about the leaf clamp, please refer to Ma et al., 2023).

#### 2.2.2 Measurement experiment of droplet physical properties

The physical properties of the droplets examined in this study include surface tension, contact angle, and droplet particle size, which were measured as follows:

Measure the surface tension coefficient (*σ_la_*) of selected deionized water using the “Wilhelmy plate method”. In addition, the contact angle measuring instrument was used to measure the contact angle of droplets on curved capsicum leaves. For static contact angle (*θ_s_*) measurement, a 3 μL droplet was placed on the leaf surface for 5 s, and its image was captured to determine the angle. For dynamic contact angle measurements, the advancing contact angle (*θ_A_*) was obtained by placing a needle near the leaf surface and injecting liquid, while the receding contact angle (*θ_R_*) was measured by withdrawing liquid from the static droplet. To calibrate droplet size, a micro-injection pump generated stable thrust on the syringe at a speed of 50 mL/h, producing droplets. The droplet size was controlled by using different types of needles, and each droplet was weighed with a precision analytical balance. Each experiment was repeated 3 times, and the average value was calculated. Finally, the droplet volume was determined using the relationship between mass and density (*m* = *ρ*·*V*).

#### 2.2.3 Measurement experiment on leaf bending deformation and substrate preparation

**Figure 3:**
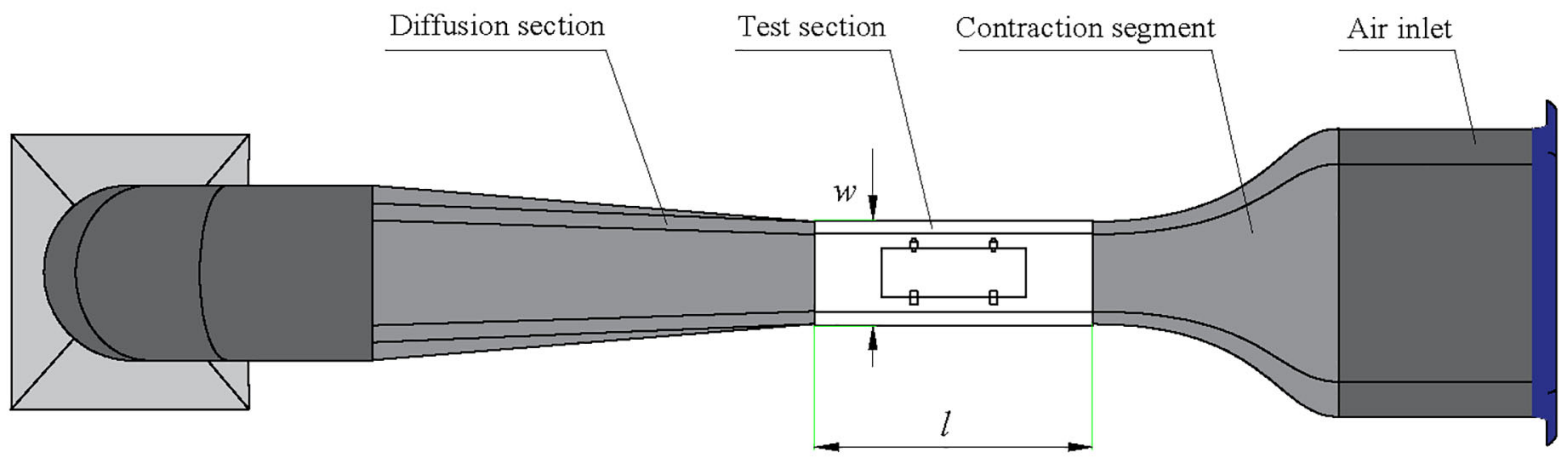
Schematic diagram of the experimental wind tunnel structure. (*l* and *w* are the length and width of the test section).

Before the experiment, the camera was calibrated. The leaf was then fixed at the center position of the wind tunnel test section using a sleeve fixture, as shown in **Figure 2**. Field measurements indicated that the wind speed reaching the leaf during long-distance air supply and spraying ranges from approximately 2.4 to 11.3 m/s. For detailed field measurement procedures and results, refer to **Supplementary File 1**. Consequently, the wind speed range selected for this experiment was 3.0 to 10.0 m/s, with increments of 2.0 m/s. The wind tunnel generated the required wind speeds during the experiment, and the overall structure of the wind tunnel system is illustrated in **Figure 3**.

By setting the desired wind speed through the speed controller, the airflow uniformly passes through the test section. This section is a rectangular channel made of transparent acrylic, measuring 1150 mm in length with a cross-sectional area of 436 × 436 mm^2^. A pitot tube was placed at the center of the test section to measure the airflow velocity in real-time with an accuracy of ± 0.1 m/s. To avoid the influence of instantaneous wind speed, the wind speed was stabilized for 5 s before collecting data on leaf motion under airflow. It was found that, although leaves might sway laterally under airflow, the primary deformation observed was bending and oscillation. This was consistent with Shao et al. (2012), as confirmed in over 80% of the test leaves. Therefore, this study focuses on leaf bending deformation as a representative measure of overall deformation, temporarily excluding the oscillation form.

For statistical purposes, the leaves need to be simplified. According to Moulia et al. (1994), the midrib of a leaf plays a crucial role in the bending deformation of the entire leaf, accounting for more than 87% of the leaf’s rigidity. Therefore, this paper uses the bending of the midrib to represent the overall leaf bending. The image digitizer software Engage Digitizer 4.1 was used to characterize the bending shape of leaves under airflow, with a set of points representing the contour of the midrib. Each midrib was marked with 50 to 70 points, at a density of 1.7 to 2.4 points/mm, with higher point density in areas of high curvature. After measuring the leaf bending deformation, the experimental data was imported into Origin 2022 and fitted using the least squares method to obtain the polynomial *f*(*x*) that characterizes leaf bending. According to the curvature definition of the curve in the plane, the curvature *κ* of each point (*x_i_*, *y_i_*) along the midrib on the leaf can be further obtained as:


(1) $$\kappa =\frac{\left|d^{2}f(x_{i})/dx^{2}\right|}{{\left\{1+{\left[df(x_{i})/dx\right]}^{2}\right\}}^{3/2}}$$

According to Equation 1, the curvature at each local position of the leaf under airflow can be determined. Given the droplet’s small size relative to the leaf, it’s assumed that the contact area between the leaf and the droplet forms a cylindrical surface. To study droplet motion on capsicum leaves with various bending deformations, curved substrates with different curvature were 3D printed to secure the leaves. The substrate is made of nylon fiber material, and the prepared substrate surface is smooth. The solid substrate size (L × W × Th) selected by the rectangular leaf size to be cut is 50 × 20 × 2 mm.

#### 2.2.4 Measurement experiment on droplet motion behavior on leaves

To study the bending characteristics of capsicum leaves without compromising their structural integrity, we employed straightforward leaf treatments. Firstly, we precisely cut between adjacent secondary veins of capsicum leaves to obtain rectangular samples measuring 6 × 18 mm. These cut leaf samples were then smoothly affixed onto custom-made curved substrates using double-sided tape. Each leaf sample was used once and replaced with new ones for subsequent experiments. The curved substrates were securely fixed on tripods using fixtures, with adjustments made to tripod height and positioning to center the leaves on the curved bases within the wind tunnel test section. Considering the relatively small size of the curved base compared to the wind tunnel test section, its impact on airflow was negligible. The syringe needle was fixed 5 mm vertically above the center of the leaf surface and connected to a micro injection pump to generate droplets of varying sizes. Droplets were gently deposited onto the leaves. During the experiment, a backlight method was employed for photography and recording. The height of the high-speed camera was adjusted to ensure the droplet was centered in the image, with the light source aligned accordingly. The camera settings were adjusted to capture images at a speed of 120 fps.

**Figure 4:**
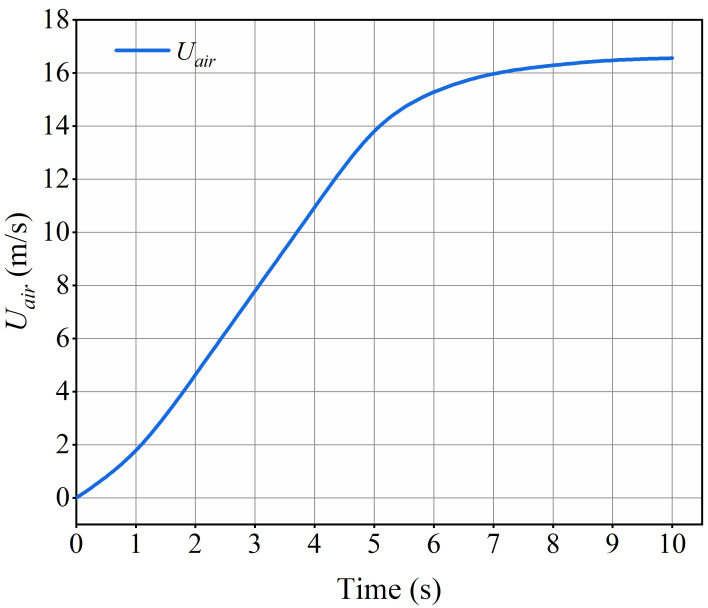
The variation of wind speed over time.

The required wind speed for this experiment was generated by the wind tunnel. By adjusting the frequency controller of the axial flow fan, we achieved a stable increase in wind speed up to the maximum value. Throughout this process, the motion of droplets was captured using a high-speed camera system. For this experiment, the frequency of the axial fan was set to 45 Hz, and the variation of wind speed from 0 to 45 Hz over time is depicted in **Figure 4**. At the start of each experiment, the fan and high-speed camera were synchronized to record real-time data on wind speed growth and images of droplet motion. The experiment continued until the droplet reached the edge of the substrate, with a maximum duration of 10 s. Each set of experiments was conducted three times under identical conditions.

To gather comprehensive insights into droplet motion on curved leaves, extracting droplet contact line information from captured images is crucial. AutoCAD 2018 drawing software is utilized for accurately outlining the droplet edge lines visible in the images. By analyzing the positions of the droplet contact lines at different points in time, key parameters that characterize droplet motion are obtained (Hu et al., 2013), such as droplet position, droplet velocity, and droplet shape variables.

## 3 Results and discussion

### 3.1 Bending deformation of leaves under airflow

**Figure 5:**
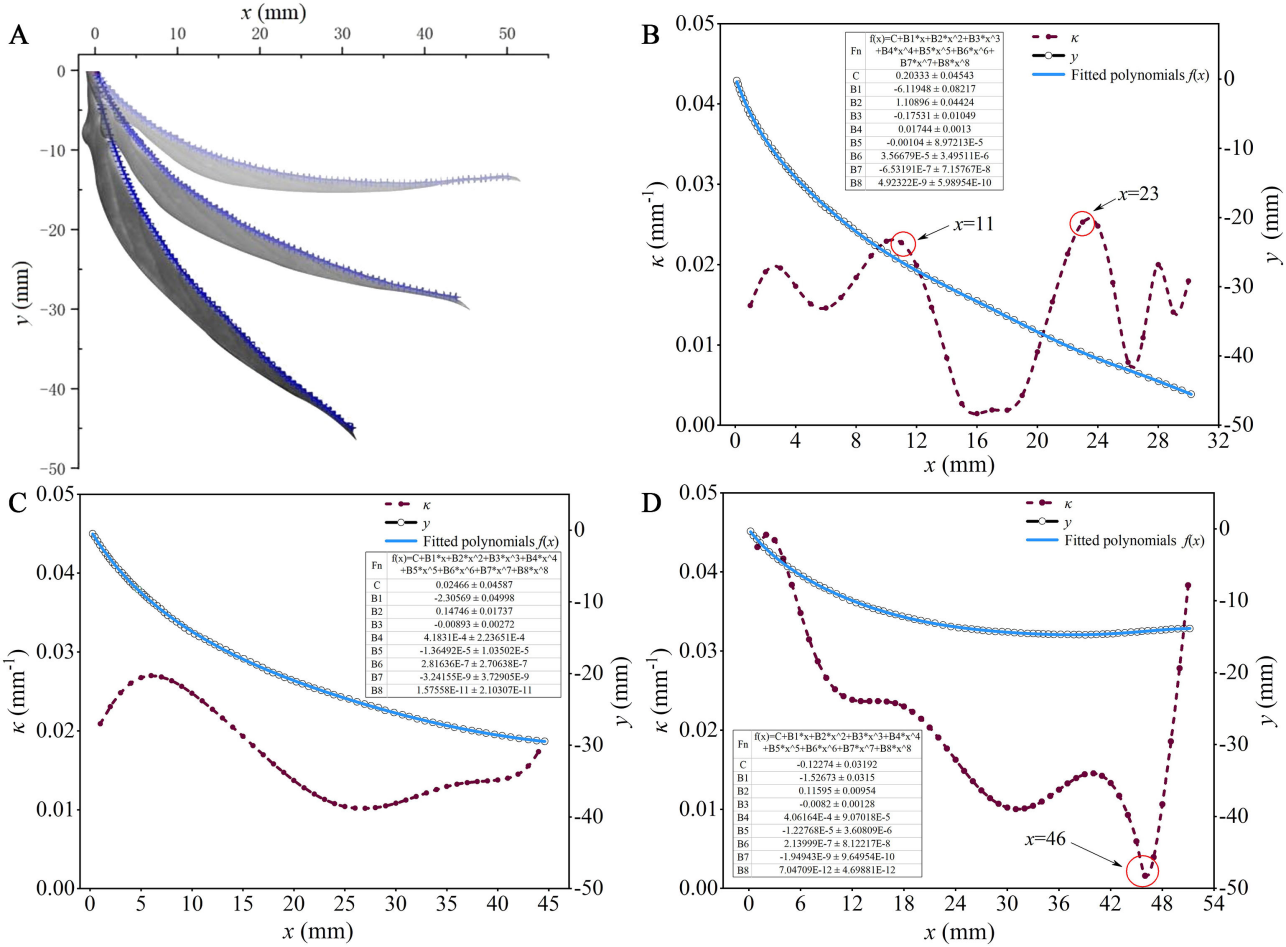
Bending deformation of leaves under different wind speeds: **(A)** Information extraction of leaf bending deformation; **(B)** A wind speed of 3.0 m/s; **(C)** A wind speed of 5.0 m/s; **(D)** A wind speed of 7.0 m/s.

To explore how the curved surface structure of the leaf affects the retention capacity of droplets, this study conducted bending deformation tests using a wind tunnel system under varying airflow velocities. The curvature was introduced to precisely digitize the leaf’s bending deformation under airflow. The coordinates of any point on the leaf’s midrib were set as the *x*-axis and *y*-axis, with the position near the petiole serving as the origin *O*. **Figure 5A** illustrates the bending deformation results of the leaves under airflow speeds of 3.0 m/s, 5.0 m/s, and 7.0 m/s, represented by color variations from dark to light. The airflow direction aligns with the positive *x*-axis.

The bending deformation of the leaf is characterized by fitting the digitized point set on the leaf midrib into a polynomial *f*(*x*). Therefore, the accuracy of the polynomial fitting becomes crucial. To determine the optimal order of the fitting polynomial, residual analysis serves as a key indicator of the fit quality. This involves generating data of the form [*x_i_*, *f*(*x_i_*) + *e*(*x_i_*)], where *x_i_* represents the true abscissa of the leaf midrib, *f*(*x_i_*) denotes the polynomial estimate of the midrib ordinate, and *e*(*x_i_*) represents the residual between the curve fitting result and the sampled point set, as illustrated in Tables in **Figures 5B–D**. The error in the sampled point set for the leaf midrib primarily arises from the thickness variations of the midrib itself, which were measured to range from 0.82 to 2.96 mm in the experimental capsicum leaves. The residuals obtained from fitting the leaf midrib curve all fall within ± 0.05 mm, indicating compliance with fitting requirements.

**Figures 5B–D** correspond to different bending deformation states of the leaves shown in **Figure 5A** under varying wind speeds, and they depict the curvature *κ* at different positions along the leaf midrib as calculated from Equation 1. In **Figures 5B**, at an airflow speed of 3.0 m/s, the overall curvature of the leaf ranges from 0 to 0.03 mm^-1^, with larger values observed in the range of *x* = 11 to 23 mm. This indicates that at lower wind speeds, bending deformation primarily occurs near the leaf tip and base, with lesser deformation in the middle of the leaf. This is due to the thinner and softer nature of the leaf near these areas, making them more susceptible to bending under the influence of wind. Conversely, the middle section of the leaf, being thicker and sturdier, offers more resistance to wind, resulting in less deformation. In **Figure 5C**, at an airflow speed of 5.0 m/s, the overall curvature of the leaf ranges from 0.01 to 0.03 mm^-1^. This suggests that the degree of bending deformation along different positions of the leaf midrib is relatively uniform. **Figure 5D** shows the results at an airflow speed of 7.0 m/s, the overall curvature of the leaf ranges from 0 to 0.05 mm^-1^, where the minimum value observed in *x* = 46 mm. This indicates that under high wind speeds, bending deformation predominantly occurs away from the leaf tip. As wind speed increases, the distribution of wind pressure on the leaf surface becomes more uniform, affecting all parts of the leaf significantly. However, due to the larger surface area near the middle and base of the leaf, greater deformation is observed in these regions. Overall, the curvature during leaf bending deformation mostly remains below 0.05 mm^-1^. Therefore, this study selected curved substrates with curvature (*κ*) of 0.01, 0.02, and 0.04 mm^-1^ to represent different bending deformations of the leaves, alongside a planar substrate (*κ* = 0) for comparison. This setup aims to explore the motion behavior of droplets on leaves with varying degrees of bending deformation.

### 3.2 Wetting characteristics of droplets on curved leaf surfaces

**Figure 6:**
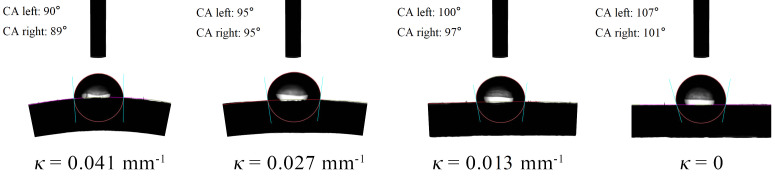
Measurement of contact angle of droplets on different curved leaves.

The wetting characteristics of droplets on a surface directly influence their ability to adhere to that surface. The static contact angle describes the angle formed by a droplet in its equilibrium state on a surface, while the dynamic contact angle refers to the angle when the droplet is in a non-equilibrium state. To explore the retention capability of droplets on curved leaf surfaces, experiments were conducted to measure the contact angles on capsicum leaves with varying curvature radii. These experiments serve as a foundational step for subsequent investigations into the motion behavior of droplets driven by airflow on curved leaves. The experimental results presented in **Table 2**. The contact angle measurement is shown in **Figure 6**.

**Table 2:** Contact angle of droplets on differently curved capsicum leaves.

*κ*/(mm^-1^)	*θ_s_*/(°)	*θ_A_*/(°)	*θ_R_*/(°)	*Δθ_CAH_*/(°)
0.041	90 ± 4	105 ± 3	65 ± 3	40 ± 6
0.027	95 ± 3	108 ± 3	69 ± 4	39 ± 7
0.013	99 ± 3	110 ± 3	73 ± 2	37 ± 5
0	104 ± 3	114 ± 3	78 ± 3	36 ± 6

The experimental results showed that as the curvature of the leaves increases, the static contact angle (*θ_s_*) of droplets on capsicum leaves tends to decrease. In other words, the greater the bending deformation of the leaf, the smaller the static contact angle between the droplet and the leaf. This finding differs from previous research on small droplets on cylindrical fibers. Brendan (1984) demonstrated that the curvature of cylindrical fibers significantly impacts droplet shape and stability. Smaller diameter cylindrical fibers lead to higher curvature effects, potentially causing droplets to become unstable or adopt non-axisymmetric shapes. In contrast, larger diameter cylindrical fibers, with their smaller curvature effect, result in more stable equilibrium shapes for droplets. McHale et al. (1999) studied the wetting behavior of small droplets on fibers with diameters ranging from 40 to 120 μm and found that the small diameter and high curvature of the fibers make droplets more likely to form wrapping structures, resulting in larger contact angles. However, for the bending deformation of leaves, the curvature of the leaves is much smaller than that of cylindrical fibers, and droplets do not form wrapping structures on the leaf surface. Additionally, an increase in leaf curvature means that droplets are more likely to contact the leaves, increasing the surface area of contact. This makes it easier for the gravity and surface tension of the droplets to balance, thereby forming a smaller contact angle.

### 3.3 The motion of droplets on curved leaf surfaces

#### 3.3.1 The critical wind speed at the beginning of droplet movement

To provide a clearer and more visual representation of droplet motion on the leaves, large droplets were selected for this experiment. The dynamic similarity between the experimental model and actual spray droplets was established using a dimensionless similarity principle, as shown in Equation 2. In field spraying, pesticides are often mixed with surfactants to lower the liquid’s surface tension, enhancing spreading and adhesion on plant leaves. The typical surface tension of such liquids ranges between 20 and 40 mN/m, with droplet diameters between 147 and 1138 μm (Butts et al., 2024). In this study, deionized water was used as the droplet liquid, with a surface tension of 72 mN/m. The Weber number (*We*) was kept constant during testing to maintain similarity with field conditions. To ensure clarity and visibility of droplet movement on the leaf, larger droplets were selected. The initial radii of the droplets were set at 1.419 mm, 1.571 mm, 1.748 mm, 1.878 mm, and 2.006 mm, respectively.


(2) $$\left\{ \begin{array}{l} We=\frac{\rho U_{a}^{2}R_{0}}{\sigma_{la}}\\ Oh=\frac{\mu }{\sqrt{\rho \sigma_{la}R_{0}}}\\ Bo_{g}=\frac{\rho R_{0}^{2}g}{\sigma_{la}},Bo_{i}=\frac{\rho R_{0}^{2}L\omega^{2}}{2\sigma_{la}} \end{array}\right.$$

Where, *ρ* is the liquid density, Kg/m^3^; *U_a_* is wind speed, m/s; *R*_0_ is the droplet radius, m; *σ_la_* is the surface tension, N/m; *μ* is the dynamic viscosity of the fluid, Pa·s; *g* is gravitational acceleration, m/s^2^; *L* is the length of the leaves, m; *ω* is the oscillation angular frequency of the leaf, rad/s, *ω*=2*πf*; *f* is the oscillation frequency of the leaf, Hz.

The small droplets in actual spray are more sensitive to viscous dissipation, while the large droplets selected for this study are more affected by gravity. Therefore, it is essential to clarify the roles of gravity and viscosity on the dynamic behavior of the droplets. Based on the definition of the dimensionless Bond number (*Bo_g_*) considering the influence of gravity, the maximum *Bo_g_* number corresponding to the droplet radius chosen in this study, *Bo_g-max_* =0.547< 1, is obtained. This indicates that the dynamic behavior of droplets is mainly influenced by surface tension, and the effects of droplet gravity is relatively small. In addition, the leaves experience some oscillation under the influence of airflow. According to the research method of Zhang et al. (2022), the oscillation frequency (*f*) of the leaves in this study is below 10 Hz. As shown in **Table 1**, the maximum leaf length (*L*) is below 0.065 m. Based on the definition of the dimensionless Bond number (*Bo_i_*) considering the influence of inertial forces, the solution is *Bo_i-max_* =7.149 > 1. This indicates that droplet behavior is influenced not only by surface tension but also by the inertial forces from leaf oscillation. This study focused on droplet size and leaf curvature, temporarily overlooking the effects of leaf oscillation. Future research will investigate how leaf oscillation influences droplet dynamics and its interaction with surface tension, enhancing our understanding of droplet-leaf interactions in pesticide spraying.

Additionally, it must be acknowledged that perfect geometric similarity between the model and reality was not fully achieved. While size similarity was matched by adjusting surface tension, this altered the contact angle. The similarity in the ratio between droplet size and leaf curvature was also not considered. Given the significant variations in contact angles and curvatures across different leaf types during field pesticide spraying, these differences were temporarily overlooked in the model. Achieving true equivalence in droplet viscosity would require significant changes in fluid properties. The dynamic similarity in this study demands that both the dimensionless *We* number and *Oh* number be equivalent. According to Equation 2, to achieve this similarity for the radius-length ratio between the selected experimental droplet and the actual spray droplet, a fluid with a kinematic viscosity approximately 10 times that of water would be ideal. However, water was used for its practicality and widespread application, leading to a deviation from the ideal *Oh* number similarity. Nonetheless, the *We* number remains constant as the primary parameter, and using water still produces meaningful results, especially for practical pesticide spraying scenarios.

**Figure 7:**
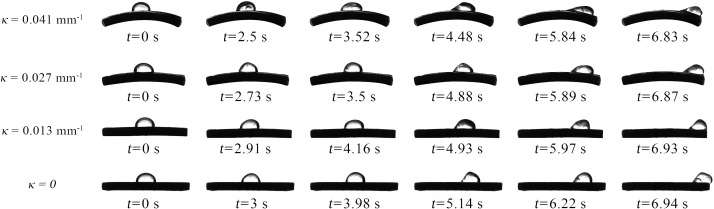
Motion process of droplets with *R*_0_ = 2.006 mm on different curved leaves.

**Figure 8:**
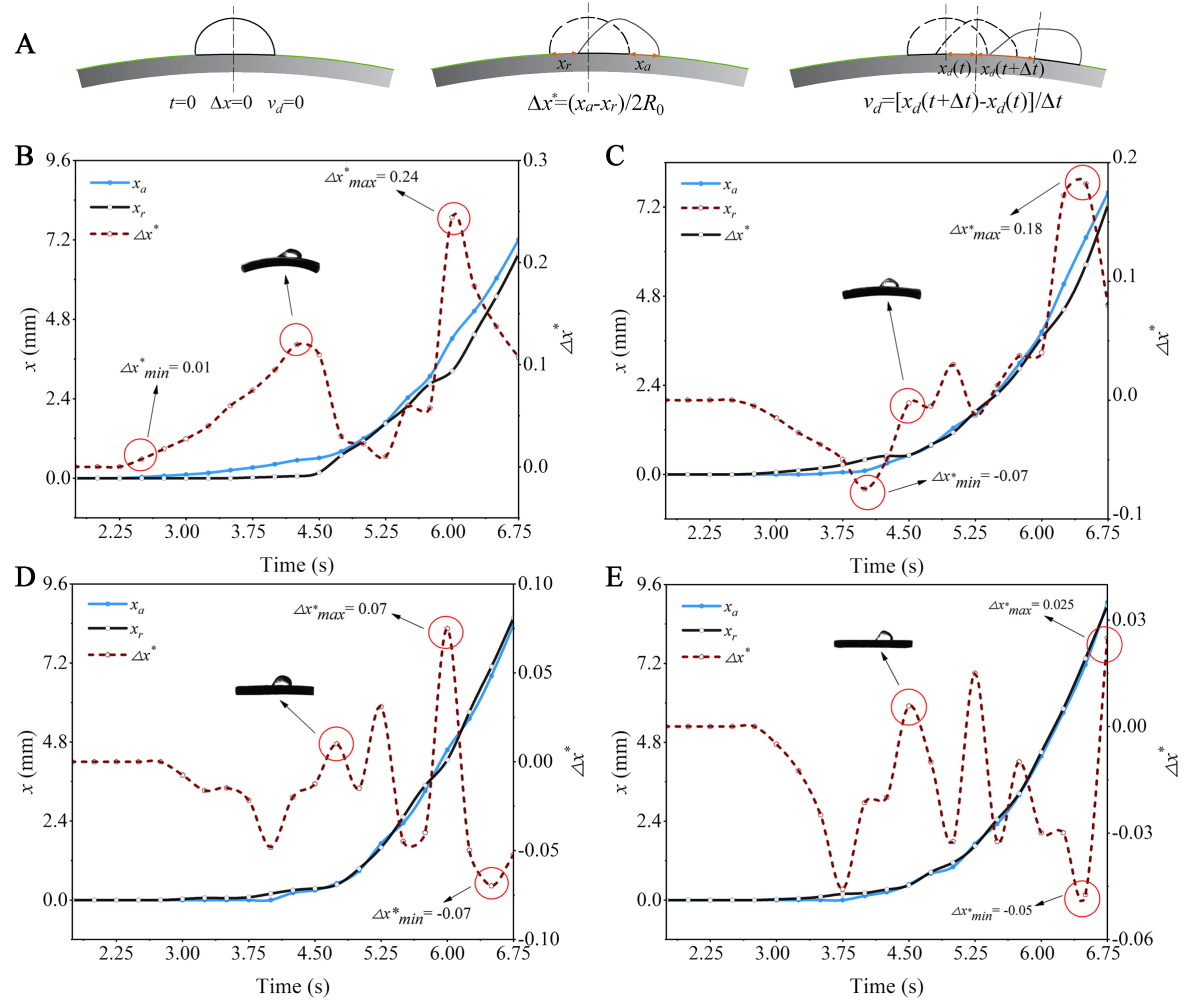
The variation of the contact line position of a droplet with *R*_0_ = 2.006 mm on different leaves over time: **(A)** Measurement methods for key characteristic parameters of droplets; **(B)** *κ* =0.041 mm^-1^; **(C)** *κ* =0.027 mm^-1^; **(D)** *κ* =0.013 mm^-1^; **(E)** *κ* =0.

Before the droplets begin to move on curved capsicum leaves, they undergo deformation and oscillation driven by airflow. **Figure 7** shows the motion behavior of a droplet with *R*_0_ = 2.006 mm on different curved leaf surfaces. As the wind speed continues to increase and exceeds a critical value, the contact line between the droplet and the leaf starts to move. This airflow velocity is defined as the critical wind speed (Wang et al., 2022). As shown in **Figure 8A**, the critical wind speed corresponds to the airflow velocity at which the first non-zero value of *x_a_* or *x_r_* occurs.

**Figure 9:**
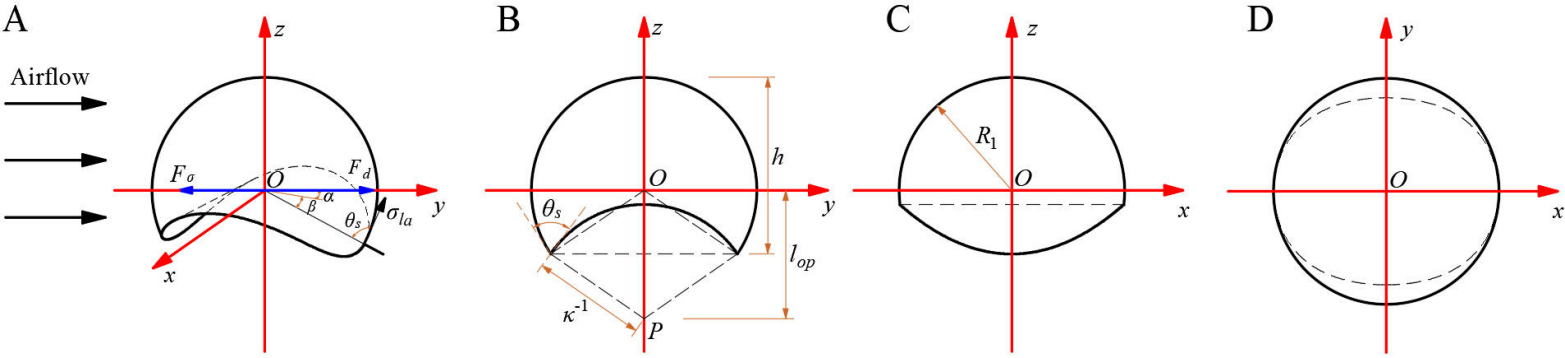
Analysis of droplet forces and its projection diagram: **(A)** Droplet force analysis; **(B)** *Oyz* plane projection; **(C)** *Oxz* plane projection; **(D)** *Oxy* plane projection.

The motion of droplets on curved leaf surfaces primarily depends on the aerodynamic force (*F_d_*) and hysteresis tension (*F_σ_*) applied to the droplets (Mahato and Mandal, 2021; Mortazavi and Jung, 2023). The hysteresis tension (*F_σ_*) is related to the forward contact angle (*θ_A_*) and the backward contact angle (*θ_R_*). To theoretically analyze the force situation of droplet motion on curved leaf surfaces, we assume that the geometric shape of the droplets on curved leaves is spherical and that the droplet shape does not change due to airflow. The only deformation considered is the local change in contact angle around the contact line. Given the small size of the droplets relative to the entire leaf, it is assumed that the contact surface between the leaf and the droplets is a cylindrical surface. The center position of the droplet is set as the origin *O* of the coordinate system, with the *y*-axis aligned with the wind direction, the *z*-axis perpendicular to the base normal, and the *x*-axis determined by the right-hand rule, as shown in **Figure 9**.

For incompressible pipeline flow with a zero pressure gradient in the vertical direction, a Reynolds number *Re*< 2100 indicates laminar flow, while *Re* > 4000 indicates turbulent flow. The range 2100< *Re*< 4000 corresponds to a transitional flow state. For both laminar and turbulent boundary layer flows along a flat channel, the boundary layer thickness (*δ*) can be approximated as follows:


(3) {δla≈5xReδtu≈0.37xRe5Re=Ua⋅xυ

Where, *Re* is the Reynolds number; *x* is the distance downstream from the starting point of the boundary layer, m; *υ* is the kinematic viscosity of the fluid, *υ* = *μ/ρ_a_*, m^2^/s.

The airflow velocity driving droplet motion in this experiment ranges from 7 to 12 m/s, and the distance *x* between the entrance of the wind tunnel test section and the droplet’s center is approximately 0.6 m. According to Equation 3, the Reynolds number at the highest wind speed is 4.93 × 10^5^, while at the lowest wind speed, it is 2.88 × 10^5^, indicating that the airflow in the wind tunnel for this experiment is turbulent. The corresponding boundary layer thickness (*δ_tu_*) at the highest wind speed is 16×10^-3^ m, and at the lowest wind speed, it is 18×10^-3^ m. Additionally, according to Equation 10, the maximum height (*h*) of the droplets placed on the leaf surface is 2.4×10^-3^ m, showing that all droplets lie within the boundary layer and are influenced by the shear flow within this region.

According to Sugiyama and Sbragaglia (2008), the shear aerodynamic force (*F_d_*) acting on a hemisphere on a solid substrate is:


(4) $$F_{d}=k\cdot (4.3\pi R_{1}\cdot \mu \cdot U_{a})$$

Where, *k* is a wall correction factor accounting for the proximity of the wall; *R*_1_ is the diameter of the fixed droplet obtained based on volume equilibrium, m.

Shear stress in fluids (*τ_s_*) is:


(5) $$\tau_{s}=\frac{\mu \cdot U_{a}}{R_{1}}$$

Numerous experiments and theoretical studies have demonstrated that, for flow inside a pipe, the frictional force (*F_s_*) generated by the fluid is proportional to the kinetic energy term (*ρ_a_U_a_* ^2^) of the fluid and the contact area (*A*) between the fluid and the wall. This relationship can be expressed as:


(6) $$F_{s}=f\cdot \frac{\rho_{a}\cdot U_{a}^{2}}{2}\cdot A$$

Furthermore, the friction coefficient (*f*) can be obtained as:


(7) {f=2τsρa⋅Ua2={16⋅Re−120.0792⋅Re−14Re<500Re≥500Re=ρaUaDμ

Where, *ρ_a_* is the fluid density, Kg/m^3^; *D* is the effective diameter of the wind tunnel test section, *D* = 4*A*_0_/*S*, m; *A*_0_ is the cross-sectional area of the wind tunnel test section, m^2^; *S* is the circumference of the wind tunnel test section, m.

Substituting Equation 7 into Equation 5, the shear stress obtained is:


(8) $$\tau_{s}=\frac{0.0792}{2}\cdot {\left(\frac{\rho_{a}U_{a}D}{\mu }\right)}^{-\frac{1}{4}}\cdot \rho_{a}\cdot U_{a}^{2}$$

By combining the above formulas and substituting them into Equation 4, the aerodynamic force (*F_d_*) acting on the droplet can be derived as follows:


(9) $$F_{d}=\frac{0.535k\cdot {R_{1}}^{2}\cdot {\rho_{a}}^{\frac{3}{4}}\cdot {U_{a}}^{\frac{7}{4}}\cdot \mu^{\frac{1}{4}}}{D^{\frac{1}{4}}}$$

According to the geometric relationship in **Figure 9**, it can be concluded that:


(10) $$\left\{ \begin{array}{l} R_{1}={\left({R_{0}}^{3}+\frac{3}{\pi }\cdot \int_{0}^{{\left({R_{1}}^{2}-{\left(\frac{1}{\kappa }-l_{op}\right)}^{2}\right)}^{\frac{1}{2}}}dx\int_{0}^{{\left(\frac{1}{\kappa^{2}}-{\left(\left(x^{2}-{R_{1}}^{2}+\frac{1}{\kappa^{2}}+{l_{op}}^{2}\right)/2l_{op}\right)}^{2}\right)}^{\frac{1}{2}}}dy\int_{-{\left({R_{1}}^{2}-x^{2}-y^{2}\right)}^{\frac{1}{2}}}^{{\left(\frac{1}{\kappa^{2}}-y^{2}\right)}^{\frac{1}{2}}-l_{op}}dz\right)}^{\frac{1}{3}}\\ A_{P}=\pi {R_{1}}^{2}\cdot \left(1-\left(\arccos\left(\left(l_{op}-\frac{1}{\kappa }\right)/R_{1}\right)/180\right)\right)+\sqrt{{R_{1}}^{2}-{\left(\frac{1}{\kappa }-l_{op}\right)}^{2}}\cdot \left(l_{op}-\frac{1}{\kappa }\right)\\ +2\cdot \int_{0}^{\sqrt{{R_{1}}^{2}-{\left(\frac{1}{\kappa }-l\right)}^{2}}}l_{op}-\frac{1}{\kappa }-y\left(x\right)dx\\ h=R_{1}+R_{1}\cdot {\left(1-\left(\frac{\sin^{2}\theta_{s}}{\kappa^{2}{R_{1}}^{2}+1-2R_{1}\cdot \kappa \cos\theta_{s}}\right)\right)}^{\frac{1}{2}}\\ l_{op}={\left({R_{1}}^{2}+\frac{1}{\kappa^{2}}-\frac{2R_{1}\cos\theta_{s}}{\kappa }\right)}^{\frac{1}{2}}\\ y\left(x\right)={\left(\frac{1}{\kappa^{2}}-{\left(\frac{\left(x^{2}-{R_{1}}^{2}+\frac{1}{\kappa^{2}}+{l_{op}}^{2}\right)}{2l_{op}}\right)}^{2}\right)}^{\frac{1}{2}} \end{array}\right.$$

Where, *h* is the height of the droplet derived from the geometric relationship in **Figure 9B**, m; *κ* is curvature of curved leaves, m^-1^; *θ_s_* is static contact angle between droplets and leaf surface, °; *A_P_* is the windward area of the droplet is derived based on the geometric relationship in **Figure 9C**, m^2^; *l_op_* is the distance between the center of the circle on the surface of a leaf and the center of mass of a spherical droplet, m; *y*(*x*) is the projection curve of the spatial curve where the droplet intersects with the leaf surface in the *Oxy* plane as shown in **Figure 9D**.

Furthermore, the functional expressions for spherical droplets and cylindrical leaf surfaces are:


(11) $$\left\{ \begin{array}{l} x^{2}+y^{2}+z^{2}={R_{1}}^{2}\\ y^{2}+{\left(z+l_{op}\right)}^{2}=\frac{1}{\kappa^{2}} \end{array}\right.$$

The hysteretic tension (*F_σ_*) is calculated by considering the force on the contact line (Fan et al., 2011). As shown in **Figure 9A**, this force is calculated by integrating the force generated by surface tension projected onto the *Oxy* plane and around the spatial curve where the droplet intersects the surface of the cylindrical leaf:


(12) $$F_{\sigma }=\oint_{L}\sigma_{la}\cdot \cos\theta \left(\alpha ,\beta \right)\cdot R_{1}\cdot \cos\beta \cdot \cos\alpha ds$$

Where, *L* is the spatial curve where the droplet intersects with the leaf surface; *θ*(*α*, *β*) is the contact angle that constantly changes along the position of the spatial curve *L*, °; *α* is the angle between the projection of the line connecting the point on the spatial curve *L* and the origin *O* on the *Oxy* plane and the *x*-axis, °; *β* is the angle between the line connecting the point on the spatial curve *L* and the origin *O* and the *Oxy* projection surface, °.

Based on Equations 9, 12, it can be concluded that as the droplet radius increases, both the aerodynamic force and hysteresis tension exhibit an upward trend. However, the increase in hysteresis tension is not as pronounced as that of the aerodynamic force. This disparity arises because hysteresis tension is mainly influenced by adhesive forces and contact angle hysteresis between the droplet and surface. While a larger radius increases the contact area, this effect is limited as hysteresis tension depends more on surface characteristics. In contrast, aerodynamic force is proportional to the upwind area, which scales with the square of the droplet radius, making even a small increase in radius significantly boost the aerodynamic force. Based on this analysis, larger droplets are more prone to sliding or detaching from the leaf surface. Additionally, as increased curvature reduces the contact area between the droplet and surface, weakening the hysteresis tension. In contrast, aerodynamic force is less dependent on contact area and more influenced by the upwind area of the droplet, making curvature have a smaller impact. As a result, higher curvature makes it easier for droplets to slide or detach.

**Figure 10:**
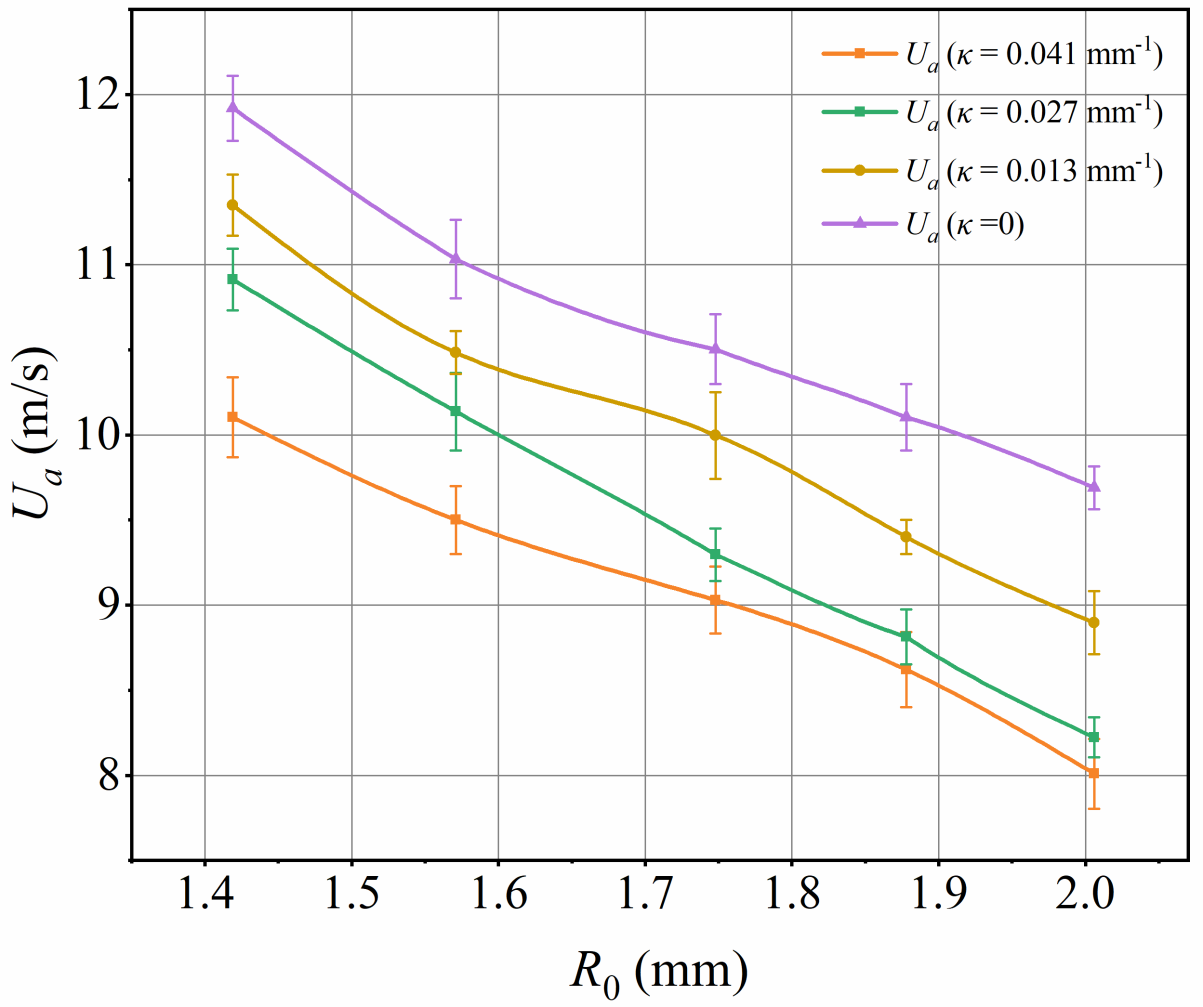
Measurement of critical wind speed at the onset of droplet motion.

Furthermore, to verify the conclusions drawn from the above theory, the critical wind speed values at which droplets begin to move on various curved leaf surfaces were measured experimentally, as shown in **Figure 10**. The error bars in the figure represent the variation in results due to repeated experiments.

As shown in **Figure 10**, the critical airflow velocity on the leaf decreases as the initial droplet radius increases, consistent with the findings of Milne and Amirfazli (2009) and Barwari et al. (2019). Experiments have shown that droplets with an initial radius of 2.006 mm require about 13.6~17.5% lower critical airflow velocity to start moving compared to droplets with an initial radius of 1.419 mm. This is because larger droplets have greater initial heights and larger front-end areas, requiring lower wind speeds to initiate movement. Equation 10 also indicates a negative correlation between the windward area (*A_p_*) and the critical wind speed. The critical wind speed not only reflects the droplets’ ability to withstand the maximum wind load on the leaf but also measures the maximum retention capacity of droplets on the leaf’s surface.

For a constant size droplet, as the curvature *κ* of the leaf increases from 0.013 to 0.041 mm^-1^, the critical wind speed at which the droplet begins to move gradually increases, indicating that the greater the leaf bending deformation, the easier it is for the droplet to move. Specifically, the critical airflow velocity for droplets on leaves with a curvature of 0.041 mm^-1^ is about 18.5~24.8% lower than that on flat leaves. This airflow velocity results from the synergistic effect of the static contact angle and contact angle hysteresis. The static contact angle determines the original shape of the droplets fixed on curved capsicum leaves: as the static contact angle increases, the contact area between the droplets and the leaves decreases, and the droplet height increases. The contact angle hysteresis determines the critical shape of the droplet at the critical airflow velocity. For the capsicum leaves used in this experiment, the static contact angle (*θ_s_*)and contact angle hysteresis (*Δθ_CAH_*) exhibit the following relationships: *θ_s_* (*κ* = 0.041 mm^-1^)< *θ_s_* (*κ* = 0.027 mm^-1^)< *θ_s_* (*κ* = 0.013 mm^-1^)< *θ_s_* (*κ* = 0) and *Δθ_CAH_* (*κ* = 0.041 mm^-1^) > *Δθ_CAH_* (*κ* = 0.027 mm^-1^) > *Δθ_CAH_* (*κ* = 0.013 mm^-1^) > *Δθ_CAH_* (*κ* = 0). Therefore, the critical airflow velocity at the beginning of the droplet movement is highest on flat leaves. It can be inferred that the critical airflow velocity at which a droplet begins to move on a leaf is positively correlated with the static contact angle and negatively correlated with the hysteresis of the contact angle. This is exactly opposite to the conclusion drawn by Wang et al. (2022) on a planar substrate. The analysis suggests that there is a significant difference between droplets fixed on curved leaves and those on flat surfaces before they begin to move. Before a droplet fixed on a curved leaf begins to move, its backward contact line undergoes an “*uphill*” motion, while its forward contact line undergoes a “*downhill*” motion. This behavior is not observed in droplets moving on a flat surface. When a droplet on a curved leaf is subjected to airflow, it oscillates, and its center of mass will not remain directly above the leaf. As the droplet’s center of gravity shifts, gravity will positively influence its movement, thus affecting the critical wind speed at which the droplet begins to move.

#### 3.3.2 The motion behavior of droplets on different curved leaf surfaces

The motion of droplets can be quantified by their position (*x_drop_*), and velocity (*v_drop_*). The value of *x_drop_* (t) is determined by the midpoint position of the arc length between the forward and backward contact lines. The velocity (*v_drop_*), is obtained by numerically differentiating *x_drop_* (t) over time (*v_drop_* = d*x_drop_*/dt), using a time interval of 0.25 s. When the droplet begins to move forward, the speed of the forward and backward contact lines cannot be consistent, resulting in different degrees of elongation of the droplet on leaves with different curvature. **Figure 8** illustrates the motion behavior of a droplet with an initial radius *R*_0_ = 2.006 mm on different curved capsicum leaves. *x_a_* represents the displacement of the droplet forward contact line relative to the initial position, while *x_r_* represents the displacement of the droplet backward contact line relative to the initial position, both expressed in arc length. (*x_a_* - *x_r_*)/2*R*_0_ is defined as the dimensionless shape variable of the droplet. The measurement method for the key characterization parameters of droplet motion is shown in **Figure 8A**.

**Figure 8B** shows that when a droplet with an initial radius *R*_0_ = 2.006 mm moves on a leaf with a curvature *κ* = 0.041 mm^-1^, the droplet dimensionless shape variable (Δx*) ranges from 0.01 to 0.24. When a droplet moves on a leaf with this curvature, it produces the largest shape variable. Overall, the greater the curvature of the leaf, the larger the droplet shape variable. This is because the gravity of the droplet itself generates additional tension, making it easier for the droplet to elongate along the bending direction. Different curved leaf surfaces affect the contact angle distribution of droplets, leading to varying forward and backward contact angles, and thus differing degrees of elongation along the curved leaf surface. In the context of plant protection spray processes, greater bending deformation of the leaf results in more significant droplet elongation. This can cause multiple droplets fixed on the leaf to converge into larger droplets, increasing the risk of droplet loss. When the curvature of the leaf is *κ* ≤ 0.027 mm^-1^, the minimum droplet dimensionless shape variable shows a negative value. This is because the droplet backward contact line is more likely to move on a leaf with a smaller curvature.

#### 3.3.3 The motion behavior of droplets of different sizes on leaves

**Figure 11:**
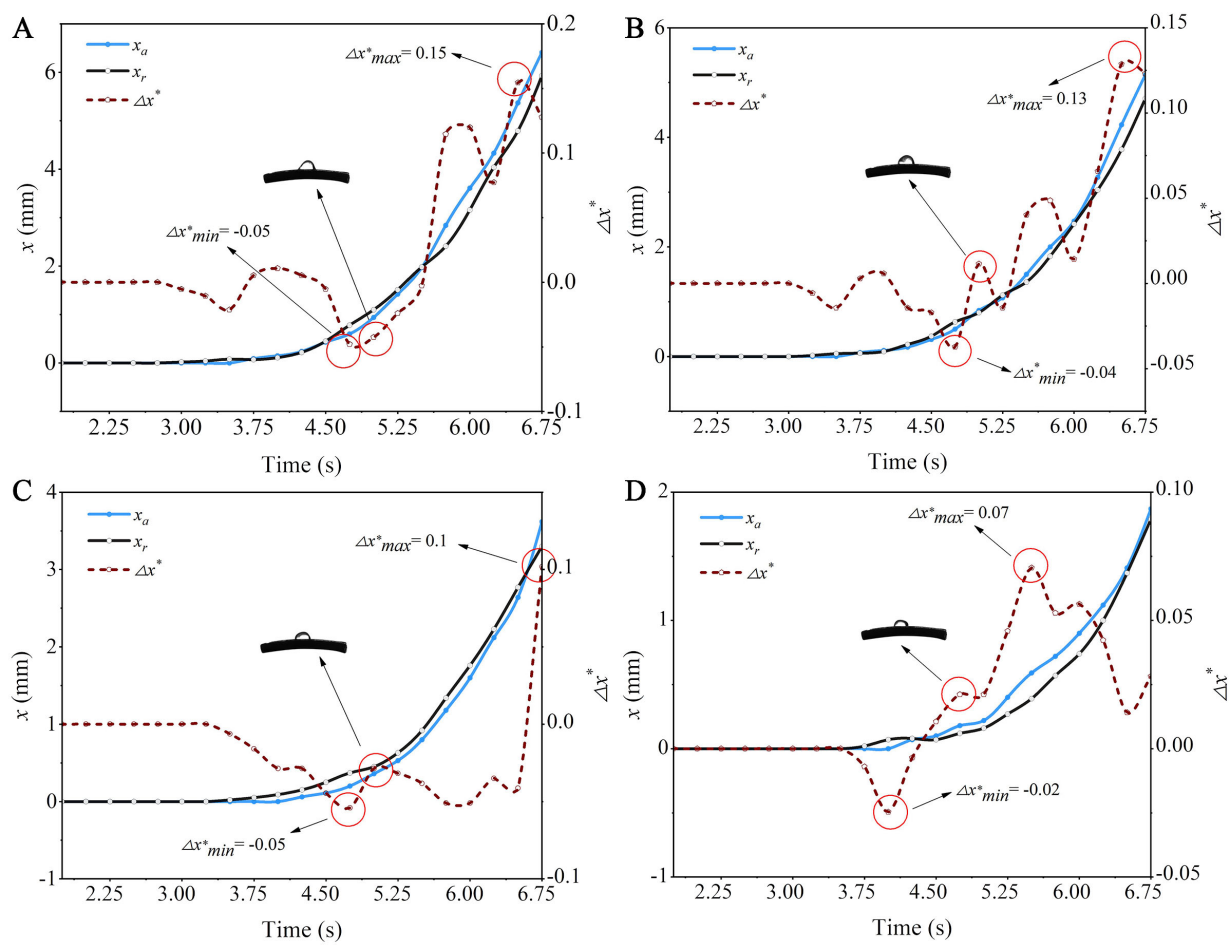
The variation of contact line positions of droplets of different sizes on a leaf with *κ* = 0.02 mm^-1^ over time: **(A)** *R*_0_ = 1.878 mm; **(B)** *R*_0_ = 1.748 mm; **(C)** *R*_0_ = 1.571 mm; **(D)** *R*_0_ = 1.419 mm.

The size of droplets affects the balance between droplet hysteresis tension and aerodynamic forces. Large droplets have a greater mass and are more challenging to move under aerodynamic driving; however, due to their larger windward area, they are subjected to greater aerodynamic forces. This equilibrium relationship impacts the shape and velocity of droplets on the surface. According to Equation 10, it is confirmed that the larger the droplet radius (*R*_1_), the smaller the corresponding hysteresis tension, and consequently, the lower the wind speed required to initiate droplet movement. This relationship is further validated and quantified through experiments. When the curvature of the leaf is constant, the motion behavior of droplets on the curved capsicum leaf, as the initial radius of the droplet increases, is shown in **Figure 11**.

**Figure 8C** shows the motion process of a droplet with an initial radius *R*_0_ = 2.006 mm on a leaf with a curvature *κ* =0.027 mm^-1^, with the droplet dimensionless shape variable (Δx^*^) ranging from -0.07 to 0.18. Comparison of **Figure 11** reveals that droplets with an initial radius *R*_0_ = 2.006 mm exhibit the largest deformation when moving on leaves with a curvature *κ* =0.027 mm^-1^, indicating that larger droplet sizes correspond to greater deformations. The analysis suggests that the larger the droplet, the more its contact line with the leaf surface expands, increasing the contact area between the droplet and the leaf, thereby raising the droplet shape variable. Overall, for droplets of different sizes, the initial droplet dimensionless shape variables are all negative, which results from the droplet backward contact line starting to move first.

#### 3.3.4 The velocity changes of droplets over time

**Figure 12:**
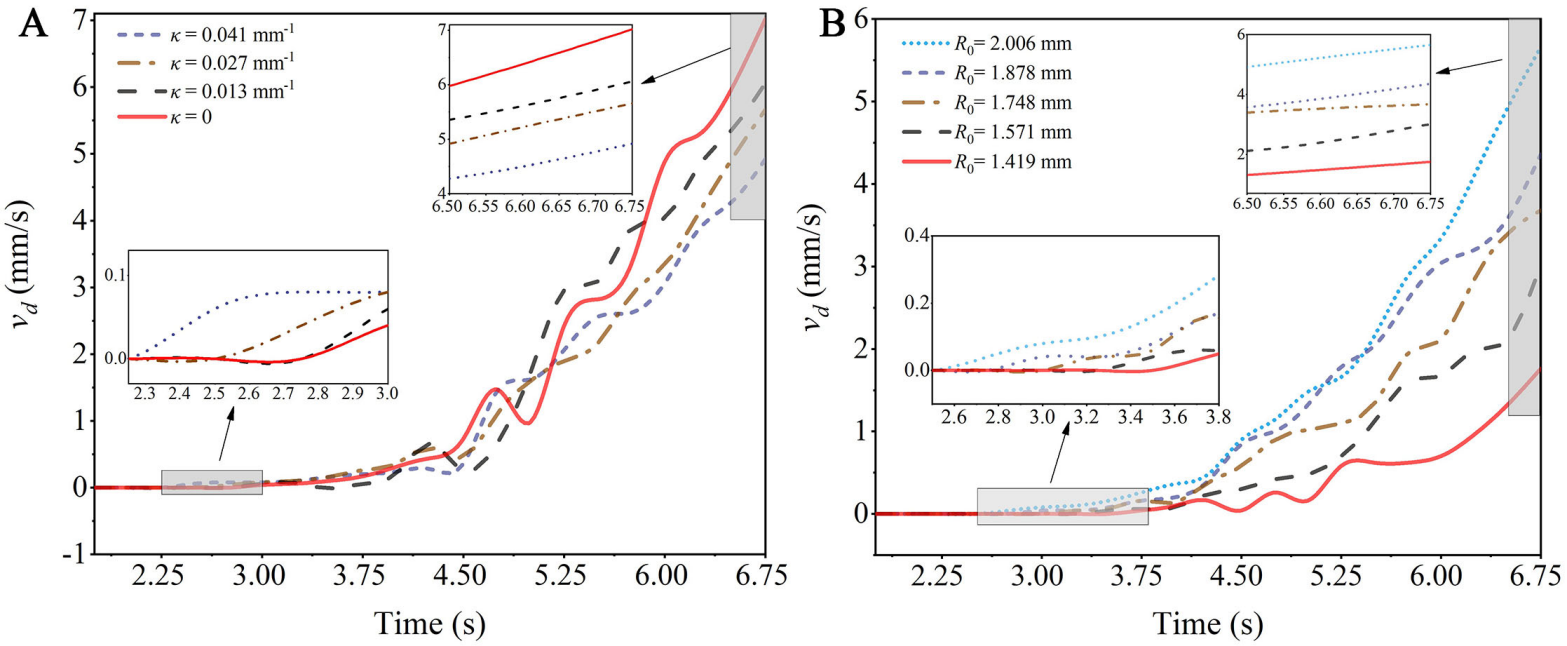
Schematic diagram of the velocity changes of droplets over time: **(A)** Leaves with different curvatures; **(B)** Droplets of different sizes.

**Figure 12A** illustrates the velocity changes of a droplet with an initial radius *R*_0_ = 2.006 mm moving on capsicum leaves with varying degrees of curvature over the test time span of 0 to 6.75 s. It is evident that the greater the curvature of the leaf, the earlier the droplet begins to move, reinforcing the conclusion that the curvature of the leaf is negatively correlated with the critical wind speed of the droplet. In general, the droplet moves with near-uniform acceleration on the curved leaf, Overall consider the accelerated motion of the droplet on curved leaves as uniformly accelerated motion, and reaches the velocity maximum at 6.75 s, the acceleration of the droplets on the leaf with curvature of 0.041 mm^-1^ is about 30% lower than that on the flat leaf. This indicates that while droplets are more prone to movement on leaves with greater bending deformation, their movement speed is relatively low, and the acceleration is also relatively modest.

**Figure 12B** shows the velocity changes of droplets of different sizes on a leaf with a curvature *κ* =0.027 mm^-1^ over the test time span of 0 to 6.75 s. It can be observed that larger droplets begin to move earlier, confirming the conclusion that droplet size is negatively correlated with the critical wind speed of the droplet. Overall, droplets of different sizes accelerate on the leaf, reaching their maximum velocity at 6.75 s. The acceleration of the droplets with an initial radius *R*_0_ = 1.419 mm is about 68.9% lower than that of droplets with an initial radius *R*_0_ = 2.006 mm. This indicates that larger droplets are more prone to movement on the leaves, and their movement speed is relatively faster with a more significant increase.

## 4 Conclusion

This study utilized wind tunnels and high-speed camera systems to obtain the bending deformation of capsicum leaves under airflow, quantified the bending deformation of the leaves through curvature *κ*. The experimental results indicate that at low wind speeds, bending deformation primarily occurs near the petiole and the tip of the leaf. At high wind speeds, the deformation shifts to areas farther from the leaf tip. Overall, the curvature *κ* during leaf bending deformation is predominantly below 0.05 mm^-1^.

Studied droplet behavior on curved capsicum leaves and developed a mechanical analysis model for droplet motion, which proved that the difficulty of droplet motion is negatively correlated with particle size and leaf curvature. The results of the critical wind speed measurement test show that the difference in critical wind speed between different particle sizes can reach 17.5%, and between droplets on various curved leaves varies by about 24.8%. Considering spray droplet parameters and bending deformation of the leaf aids in understanding maximum droplet retention capacity during air-assisted spraying.

During droplet movement, both the curvature of the leaf and the droplet size significantly influence the droplet shape variable. Within the test duration, the maximum dimensionless shape variable on various curved leaves reaches 0.24, and the acceleration can differ by about 30%. For droplets of different sizes, the maximum dimensionless shape variable is 0.18, the acceleration can differ by about 68%. This distinct motion behavior of droplets on different curved leaves helps to understand droplet-leaf interactions and provides new insights for optimizing pesticide application.

## Data Availability

The datasets presented in this study can be found in online repositories. The names of the repository/repositories and accession number(s) can be found in the article/**Supplementary Material**.
