# Supplementary material for: Motion behavior of droplets on curved leaf surfaces driven by airflow
Source: Front Plant Sci. 2024 Oct 15;15:1450831. doi: 10.3389/fpls.2024.1450831 (PMC11518756; doi:10.3389/fpls.2024.1450831)
Supplement: Supplementary file 1 [file DataSheet1.pdf]

Supplementary Material for

Motion behavior of droplets on curved leaf surfaces driven by airflow

Zhou-Ming Gao, Wei Hu, Xiao-Ya Dong*, Xiao-Yuan Zhao, Song Wang, Jian Chen, Bai-Jing Qiu*

Key Laboratory of Modern Agricultural Equipment and Technology, Ministry of Education, Jiangsu University, Zhenjiang, Jiangsu, 212013, China

Key Laboratory of Plant Protection Engineering, Ministry of Agriculture and Rural Affairs, Jiangsu University, Zhenjiang, Jiangsu, 212013, China

- Wind speed measurement experiment in the process of long-distance air supply spray

The wind speed measurement test in a solar greenhouse in Shouguang City, Weifang City, Shandong Province, China on October 18, 2023. The experimental instruments mainly include the single suspension crawler type independent air supply spray machine (see Lin et al., 2021. Development and test of an autonomous air-assisted sprayer based on single hanging track for solar greenhouse, Crop Protection, 142, 105502.), three-dimensional ultrasonic anemometer, meter ruler, laptop, etc. During the test, two three-dimensional ultrasonic anemometers were used to measure the wind speed near the crops on both sides of the crop row. The *x*-axis position of the three-dimensional ultrasonic anemometer was set to be in the same direction as the wind speed in the forward direction. The wind speed values at the positions 2 m, 3 m, 4 m and 5 m away from the spray were measured in 217 s. The field test site is shown in Figure S1. The wind speed measurement test results are shown in Figure S2.

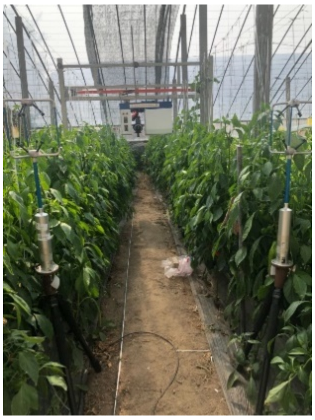

Figure S1. Field testing site

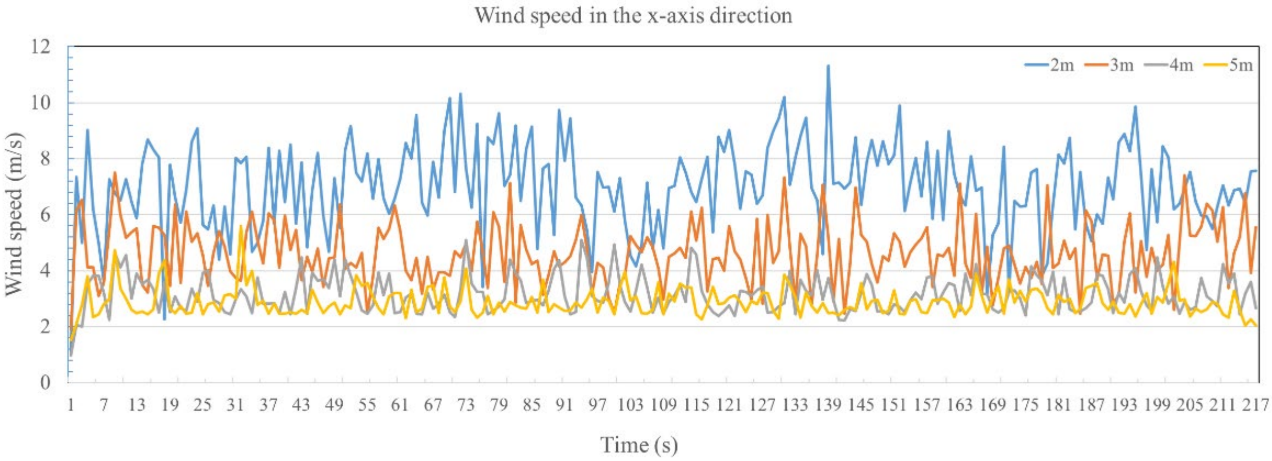

Figure S2. Test results

According to the wind speed measurement test results, as the distance from the spray gets farther and farther, the wind speed value shows a declining trend. When the distance from the spray is 2 m, the maximum wind speed reaching the crops is 11.3 m/s, and when the distance from the spray is 5 m, the minimum wind speed reaching the crops is 2.4 m/s.
